# Two new species of *Brusqeulia* Razowski & Becker, 2000 from the Neotropics, with comments on the systematic position of the genus in relation to the *Apolychrosis* Amsel, 1962 group of genera (Lepidoptera, Tortricidae, Cochylini)

**DOI:** 10.3897/zookeys.770.24281

**Published:** 2018-07-03

**Authors:** Jose V. Pérez Santa-Rita, Joaquin Baixeras

**Affiliations:** 1 Cavanilles Institute of Biodiversity and Evolutionary Biology, University of Valencia, Calle Catedrático José Beltrán, 2, 46980-Valencia, Spain

**Keywords:** *Brusqeulia araguensis*, *Brusqeulia yunkensis*, Euliina, South America, subpapillar sclerite, systematics, taxonomy

## Abstract

Two new species of the neotropical genus *Brusqeulia* Razowski & Becker, 2000, are described and illustrated: *B. yunkensis* Pérez Santa-Rita & Baixeras, **sp. n.** from Bolivia and *B. araguensis* Pérez Sant-Rita & Baixeras, **sp. n.** from Venezuela. The systematic position and diagnostic characters of the genus are reviewed, resulting in the synonymy of *Pinhaisania* Razowski & Becker, 2000, with *Brusqeulia*, and the combination *B. crispula* (Razowski & Becker, 2000), **comb. n.** New characters of the female genitalia are discussed.

## Introduction

Tortricoidea are a monophyletic and rather homogeneous superfamily of Lepidoptera that includes the single family Tortricidae, containing more than 10,800 described species (Gilligan et al. 2014). Nineteen tribes organised in three subfamilies are currently recognised. The tribe Cochylini includes more than 2,150 species assigned to 246 genera, of which 1,041 species and 169 genera belong to the subtribe Euliina (Gilligan et al. 2014). Euliini was proposed by Kuznetsov and Stekolnikov (1977) as the monophyletic sister group (subtribe Euliae) to Cochylini (Cochyliae), with the former subsequently elevated to tribal level by Powell (1986). Molecular evidence (Regier et al. 2012) strongly supports the monophyly of Cochylini s. str., but leaves Euliini as a paraphyletic taxon. Cochylini has nomenclatorial priority but for practical reasons, it has been recommended that the two hypothetical subtribes Euliina and Cochylina be retained (Gilligan et al. 2014, Gilligan and Brown 2016, Pérez Santa-Rita and Baixeras 2017). We follow that convention in this paper.

Horak (1998) suggested that the loss of forewing CuP, the loss of uncus and gnathos in the male genitalia, and the ill-defined signum in female genitalia are diagnostic characters for Cochylina. Monsalve et al. (2011) found a two-bristled female frenulum is also a common condition in the group. However, these characters are reductive trends shared with Euliina, not true autapomorphic characters (Brown and Powell 1991). The presence of one or more large non-deciduous, laterobasally attached, aciculate, capitate cornuti with a microdenticulate vesica is also evidence of the relationship between the two subtribes (Anzaldo et al. 2014). Extreme morphological variation, especially in the genitalia, contributes to the difficulty of defining Cochylini (Pérez Santa-Rita and Baixeras 2017) and makes it difficult to establish boundaries between the taxa. In fact, more than half of the genera of the subtribe Euliina are monotypic, mostly from the Neotropics. In this taxonomic and morphological scenario, how Cochylina is nested within Euliina remains unresolved.

The Neotropical genus *Brusqeulia* Razowski & Becker, 2000 (originally placed in Euliina), is an interesting example of a mixture of euliine and cochyline characters that is in agreement with the transitional role which the molecular data indicate for Euliina. Brown and Powell (1991) recognised a group of genera organised around *Apolychrosis* Amsel, 1962 to be basal with respect to a well-defined *Chrysoxena* Meyrick, 1912 group of genera. Brown and Adamski (2003) detailed the systematic composition of the *Apolychrosis* group, one of the few groups of Euliina for which a phylogeny has been proposed. The study of two new species of *Brusqeulia* shows evidence of the connection of this genus with the *Apolychrosis* group and allows speculation regarding relationships within Cochylini.

## Materials and methods

Specimens were obtained by light trapping in Bolivia (different localities) by the second author (JB) and from museum collections (listed below). See supplementary file 1: material_examined.xls for a detailed account of the material examined. Dissection procedures follow Robinson (1976) and Zlatkov (2011), and were performed under a Leica MZ8 stereomicroscope. Adults and genitalia were photographed using a Leica Z16 microscope, equipped with a CF500 camera and LAS 4.9 (Leica) image capture software. Z-stacks followed by extended depth of field application was extensively used to produce final images. When the level of pressure on the genitalia preparation is considered to change the shape of the valvae, the value of the thickness of the preparation is determined by the z-range during the acquisition and is indicated in Figures 2B and C. Methods for scanning electron microscope (SEM) preparation and observation follow Lincango et al. (2013). All images were edited using Photoshop CS3 (Adobe). Terminology of the genitalia follows Horak (1984) and Anzaldo et al. (2014); terminology for elements of the forewing pattern follows R. Brown and Powell (1991) as modified by Baixeras (2002). Forewing measurements were taken along a straight line from the base of the wing to the apex (including fringe). Range, mean (*x̄*), and number (n) of specimens measured are indicated throughout the text.

DNA extraction was performed from an abdomen according to NucleoSpin XS Tissue purification procedure (Macherey-Nagel Duren, Germany). COI was amplified by PCR using LepF1 / LepR1 primers (Hebert et al. 2004). The selected PCR products were purified following High Pure PCR Product Roche Purification protocol. DNA labeling was performed with BigDye Terminator v3.1 Cycle Sequencing Ready Reaction ABI PRISM (Applied Biosystems). Amplicons were sequenced by Sanger method (Sanger and Coulson 1975) in an ABI 3730 DNA sequencing equipment (Applied Biosystems). Reading and assembly of the 658 bp sequence was assisted by STADEN software Package (Staden 1999). Finally, the sequence was tested against GenBank by BLAST.

A phylogenetic analysis was conducted to determine the relative position of the newly described taxa within the *Apolychrosis* group of genera. The character matrix of Brown and Adamski (2003) was used as the starting point (i.e., 25 morphological characters), to which we added character states for the two new species of *Brusqeulia*. However, as discussed below, the group of *Punctapinella niphastra* was finally excluded from the analysis in order to improve resolution. We converted two of the 25 original characters from binary to multi-state: the vesica of the phallus in male genitalia and the sterigma in female genitalia [characters 21 and 22, respectively, in Brown and Adamski (2003). Character 21 adds a character state “2” referring to the presence of two types of cornuti (aciculate + microspinulate). Character 22 adds a character state “2” referring to the sterigma complex. Brown and Adamski (2003) assumed that the ductus seminalis (Character 25) originated from the ductus bursae in *Eubetia* Brown, 1999. However, re-examination of this character (J. Brown, personal communication) confirmed that the intersection of the ductus seminalis and the bursa copulatrix is really in the cervix and consequently part of the corpus bursae. This character state has been changed accordingly in the data matrix. The data matrix is included as supplementary file 2: character_matrix. Characters were coded and subjected to parsimony analysis using the TnT version 1.5 (Goloboff and Catalano 2016). Each character was added one by one in the taxa analyzed. We used “Traditional Search” method with 1000 random addition sequences (RAS), each with the option tree bisection reconnection (TBR) branch-swapping (Goloboff et al. 2008).

### Abbreviations

**MNKM** Museo de Historia Natural Noel Kempff Mercado, Universidad Autónoma Gabriel René Moreno, Santa Cruz de la Sierra, Bolivia.

**USNM** National Museum of Natural History, Smithsonian Institution, Washington D.C., United States.

**ICBiBE** Institut Cavanilles de Biodiversitat i Biologia Evolutiva, Universitat de València, Spain.

## Taxonomy

### Brusqeulia

Taxon classificationAnimaliaLepidopteraTortricidae

Razowski & Becker, 2000

Brusqeulia Razowski & Becker, 2000, SHILAP Revista de Lepidopterología 28: 386; type-species: Brusqeulia sebastiani Razowski & Becker, 2000
Pinhaisania Razowski & Becker, 2000, SHILAP Revista de Lepidopterología 28: 387; type-species: Pinhaisania crispula Razowski & Becker, 2000 – **syn. n.**

#### Diagnosis.

**Figure 1.**
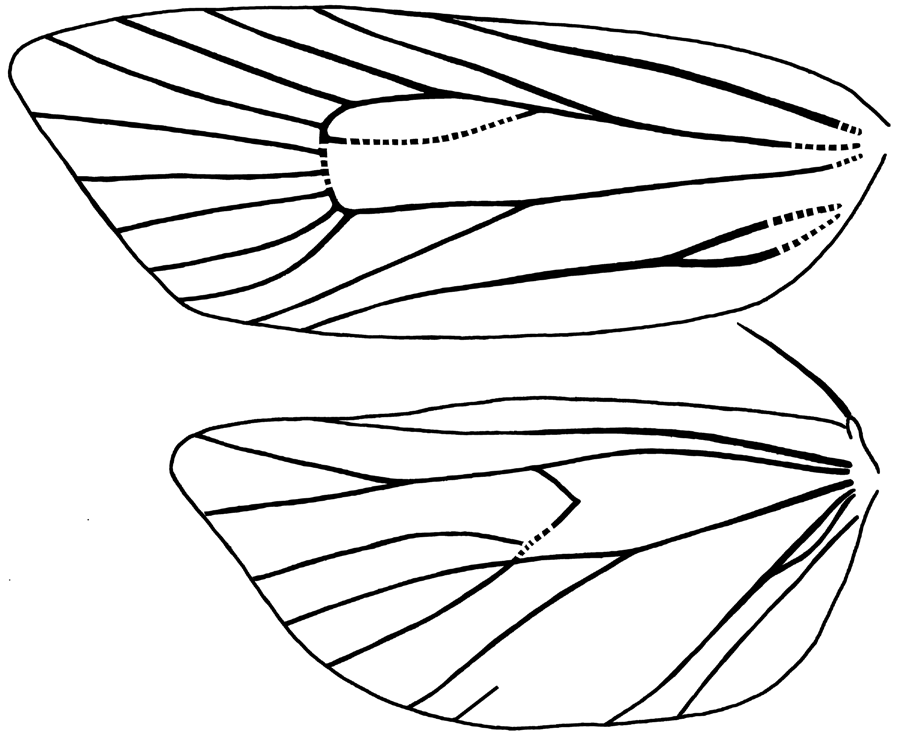
Venation in *Brusqeulia yunkensis*. Based on slides JBA20815 and JBA20836. Dashed lines indicate obsolescent veins.

Venation typically for Cochylina (Fig. 1). Forewing (based on two slides) without costal fold; discal cell ca. 0.6 times length of wing, no M–stem, chorda obsolescent ca. 0.25 times length of wing, cross veins vestigial; all veins present except CuP; R_4_ to the costa near apex, R_5_ to the termen; distances between pairs of veins R_5_–M_1,_ M_1_-M_2_ and M_2_–M_3_ on termen similar; distances between M3–CuA_1_, CuA_1_–CuA_2_ and CuA_2_–1A+2A similar; CuA_2_ opposite on discal cell ca. 0.3–0.5 the distance between R1 and R_2_ on the cell, approximately coincident with the point where the chorda meets Rs; anal loop ca. 0.3 times length of 1A+2A. Hindwing with Sc+R_1_ somewhat parallel to Rs basally, length ca. 0.8 times length of wing; M_1_ and Rs stalked in basal half; M_2_, M_3_ and CuA_1_ obsolescent basally; M_3_ and CuA_1_ connate; CuA_2_ well developed, CuP reduced, present only in distal portion; 1A+2A and 3A developed, anal loop ca. 0.4 times length of 1A+2A. Frenulum in males with one single bristle, three bristles in females. Male genitalia with transtilla broad and well developed; gnathos as two arms fused distally forming a short process, resulting in a plicate terminal plate; characteristic valva, elongate; cucullus with a more or less developed disc of hair like scales; sacculus with a free terminal process. Phallus with two distinctive sets of non-deciduous cornuti, one set as a ventral band of rather large aciculate cornuti basally attached; a second set in the inner part of vesica formed by microspinulate cornuti. Female genitalia with lobular lamella antevaginalis and postvaginalis; ventral spinous subpapillar sclerite on the 8-9 intersegmental membrane at the level of the ventral lobes of the anal papillae.

#### Diversity and distribution.

Fifteen species have been described from Brazil and one from Ecuador (Razowski and Becker 2000, 2011). To that we add one species from Bolivia and another from Venezuela. Given the broad geographical and elevational range (from near sea level to ca. 2000 m), we suspect that *Brusqeulia* includes additional undiscovered species.

## Checklist of species

*Brusqeulia araguensis* Pérez Santa-Rita & Baixeras, 2018 – **sp. n.**

*Brusqeulia atrocentra* Razowski & Becker, 2011

*Brusqeulia atrograpta* Razowski & Becker, 2011

*Brusqeulia baeza* Razowski & Becker, 2011

*Brusqeulia bonita* Razowski & Becker, 2011

*Brusqeulia caracagena* Razowski & Becker, 2011

*Brusqeulia ceriphora* Razowski & Becker, 2011

*Brusqeulia costispina* Razowski & Becker, 2011

*Brusqeulia crispula* (Razowski & Becker, 2000) (*Pinhaisania*) – **comb. n.**

*Brusqeulia guaramiranga* Razowski & Becker, 2011

*Brusqeulia jacupiranga* Razowski & Becker, 2011

*Brusqeulia monoloba* Razowski & Becker, 2011

*Brusqeulia sebastiani* Razowski & Becker, 2000

*Brusqeulia signifera* Razowski & Becker, 2000

*Brusqeulia tineimorpha* Razowski & Becker, 2011

*Brusqeulia tripuncta* Razowski & Becker, 2000

*Brusqeulia uncicera* Razowski & Becker, 2011

*Brusqeulia yunkensis* Pérez Santa-Rita & Baixeras, 2018 – **sp. n.**

## 

### Brusqeulia yunkensis sp. n.

Taxon classificationAnimaliaLepidopteraTortricidae

http://zoobank.org/1BFCD272-BD76-4312-9804-7E65744802E8

Figures 2 , 4A, C

#### Type material.

***Holotype***: ♂, Bolivia, Santa Cruz Department, Florida Province, Pampa Grande Municipality, locality of Hueco de la Pascana, 1575 m, 18°7.09'S; 64°3.58'W, 25 Jan 2011, J. Baixeras, A. Valdivia and G. Fernández (MNKM).

***Paratypes***: (15♂, 6♀). Bolivia: Santa Cruz Department, Florida Province, Mairana Municipality, locality of Yunga de Mairana, Rasete, 2000 m, 18°04'S; 63°54'W, 4 Nov 2005 (6♂, 3♀), J. Baixeras, A. Valdivia and I. García (GS USNM 124290, USNM 124291); Yunga de Mairana, ca. Bosque de Helechos, 2150 m, 18°03'S; 63°55'W , 02 Nov 2005 (1♂), J. Baixeras, A. Valdivia and I. García (GS 20724); locality of Pampa Grande, Hueco de la Pascana, 18°7.09'S; 64°3.58’W, 10 Nov 2001 (1♂), A. Valdivia and J. Baixeras; Pampa Grande, La Hoyada, 1600 m, 17°57'S; 64°06'W , 07 Nov 2005 (1♂, 1♀), J. Baixeras, A. Valdivia and I. García (GS 20727, 20728); Pampa Grande, Agua Clarita, 1554 m, 17°56.74'S; 64°7.97'W 27 Jan 2011 (5♂, 2♀), J. Baixeras, A. Valdivia and G. Fernández; Pampa Grande, El Milu, 1534 m, 17°59.36'S; 64°3.23'W, 28 Jan 2011 (1♂), J. Baixeras, A. Valdivia and G. Fernández. Paratypes deposited in MNKM, USNM, and ICBiBE.

#### Material examined not included in the type series.

Bolivia: Santa Cruz Department, Florida Province, Mairana Municipality, locality of Yunga de Mairana, Rasete, 2000 m, 18°04'S; 63°54'W, 4 Nov 2005 (1♂, 1♀), J. Baixeras, A. Valdivia and I. García (GS JBA20684, JBA20815, SEM stub JBA193); Pampa Grande, Agua Clarita, 1554 m, 17°56.74'S; 64°7.97'W 27 Jan 2011 (2♂), J. Baixeras, A. Valdivia and G. Fernández (GS JBA20836, JBA20844, JBA20864). Deposited in ICBiBE.

#### Molecular characterisation.

We were able to obtain partial COI sequence data (i.e., the DNA barcode) for a single specimen (GENBANK accession number MG951753), and comparison of the sequence against Genbank did not render any useful information. Interestingly, sequencing of a second sample revealed the presence of DNA related to the entomopathogenic trypanosomatid genus *Crithidia* Léger, 1902 (phylum Euglenozoa; GENBANK accession number MH118295).

#### Diagnosis.

**Figure 2.**
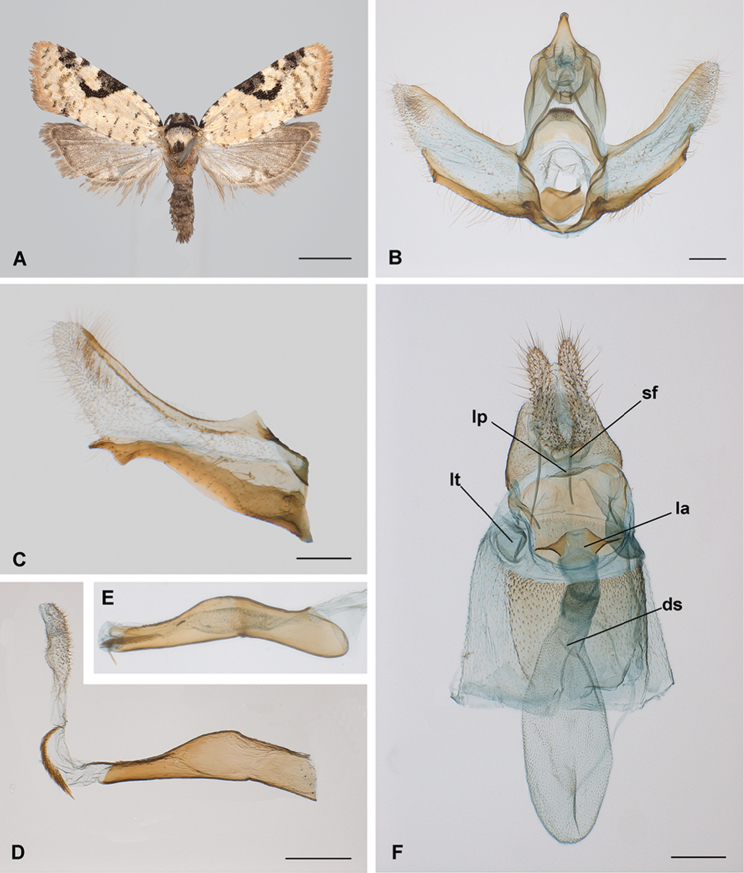
Morphological characters of *Brusqeulia yunkensis* sp. n. **A** habitus (Paratype, male, Bolivia, Santa Cruz, Pampagrande, 25 January 2011, ICBiBE) **B** male genitalia, phallus removed (GS JBA20728, z = 161 µm) **C** male genitalia left valva completely extended (GS JBA20684, z = 83 µm) **D** phallus with everted vesica (caecum removed, GS JBA20844) **E** phallus uneverted (GS JBA20728, same scale as D) **F** female genitalia (GS JBA20727). Abbreviations. ds: ductus seminalis connection to the bursa; la, lamella antevaginalis; lp, sclerite on the lamella postvaginalis; lt, lateral pocket; sf, ventral spinous field of segment 8. Scale bars: 2 mm (**A**); 200 µm (**B, C, D, F**).

The habitus of *B. yunkensis* (Fig. 2A) does not ensure discrimination from similar species of *Brusqeulia* (e.g., *B. baeza* or *B. uncicera*) or species of the closely related genus *Limeulia* Razowski & Becker, 2000. An examination of the available literature suggests that the crescent-shaped blotch of the forewing costa is present in all the species of the genus *Brusqeulia* and related genera. The distinctive characters at the species level are associated with the male and female genitalia. *Brusqeulia yunkensis* can be distinguished by the unusual configuration of the transtilla in males (Fig. 2B). The transtilla is well developed in most species of *Brusqeulia* and in associated genera, but a transtilla projecting posteriorly into a flat spinulous area is found only in *B. crispula* and presumably in *B. monoloba*. However, in *B. yunkensis* the spinulous area occupies ca. 0.2 of the total length of the transtilla, whereas in *B. crispula* and *B. monoloba* it occupies ca. 0.3 of the total length. The impression is a longer, more protruding transtilla in *B. yunkensis* than in the other two species. *Brusqeulia crispula* has a distinctive pillous disc on the cucullus that is present but only weakly developed in *B. yunkensis*; no disc is apparent in *B. monoloba*. There is wide variation in the development of the uncus in *Brusqeulia* species, from thin projections, as in *B. bonita*, *B. baeza*, and *B. araguensis* sp. n., to relatively broad finger-like projections, as in *B. crispula* and *B. tripuncta*. *Brusqeulia yunkensis* has a moderate development similar to that of *B. teneimorpha* and *B. guaramiranga*. A rugous spatulate projection of the gnathos has never been described in species of the group and could be a unique character. An inward curved sacculus distally projecting into a pointed process (Fig. 2C) is similar to that found in some species such as *B. baeza*, *B. monoloba* and even *B. crispula*, but the shape of the terminal process is diagnostic in every species of the group. Finally, the phallus of *B. yunkensis* seems to be a simplified organ with respect to the typical stout structure in its relatives, more elongate and without any distal ventral process. The presence of denticles on the dorsal distal part of the phallus (Fig. 2E) together with slender terminal cornuti (Fig. 2D) has been reported only in *B. sebastiani*. The presence of microspinulation on the inner part of the vesica is unknown in other species of the genus. So far, morphological details of the females of *Brusqeulia* are limited owing to the paucity of material. The only female described is *B. caracagena*, a species for which the male is unknown. The latter can be easily distinguished from *B. yunkensis* by the ductus bursae (Fig. 2F) – extremely short in *B. caracagena*, longer and partially sclerotised in *B. yunkensis*. The spinous subpapillar sclerite (Fig. 4B and D) on the intersegmental membrane, present in *B. yunkensis*, is absent in *B. caracagena*.

#### Description.

*Head*: Vertex with long brownish scales protruding anteriorly and dorsally, fan-shaped, between antennae. Frons slightly convex covered with some whitish scales. Antennae dark brown, length ca. 0.5 as long as forewing costa, dorsally scaled, ventrally ciliated, two rows of scales per flagellomere. Palpus labialis porrect, length (all three segments combined) ca. 1.4 times diameter of compound eye, uniformly scaled; first segment short, slightly upcurved, with brown scales, second segment long, straight with mixed brown scales laterally and whitish scales dorsally, third segment short and slightly upcurved with whitish scales basally and apically and brown scales medially; opening of organ of vom Rath in apical position. Haustellum well developed. Ocelli and chaetosemata well developed.

*Thorax*: Upperside with pronotum, anterior half-part of mesoscutum, and tegulae covered by dark brown scales and posterior half-part of mesoscutum and metanotum covered by white scales; smooth-scaled including tegulae, without scales tufts. Underside, including legs, whitish, male foreleg hairpencil absent. Forewing length 5.0–6.6 mm (x̄ = 6.1; n = 19) in males, 5.8–7.3 mm (x̄ = 6.7; n = 7) in females. Forewing with typical venation of Cochylina, details described for the genus. Forewing pattern not sexually dimorphic (Fig. 2A). Forewing upperside with ground colour whitish with brownish-grey marking; most marks concentrated in costal area; system of pairs of strigulae vaguely recognisable, presumably concolourous with background, only through inter strigular dark marks; some scattered marks at basal fourth of costa; marks at level of Sc fused to produce a distinctive crescent shape brownish-grey blotch projected discally in a rather conspicuous coma-like patch confluent with R2–R3; single marks between Sc and R1, R1 and R2, R2 and R3, no marks beyond R3; some scattered grey scales between marks; striae strongly fragmented; dorsal marking ill-defined; fasciae undetectable; fringe ochreous; forewing underside uniformly brownish ochreous with some pale strigulae at radial level on the costa; overlapping area whitish. Hindwing upperside and underside, including fringe, uniformly brownish-ochreous; male costal fold absent; cubital pecten not detected.

*Abdomen*: Dorsad greyish, paler ochreous cephalad. Segment 8 unmodified. Male genitalia (based on four preparations; Fig. 2B, C, D, E) with tegumen well developed, laterally straight; uncus developed, basally confluent with top of tegumen, progressively narrowed distally; socii membranous, conspicuous, moderately developed, hairy; gnathos as two arms distally fused distally into a short process, moderately expanded distally in a central spinous molar-like sclerite; transtilla broad, strong, with a distal moderately flat area densely covered by short strong spines; valva elongate, variable in shape (Fig. 2B and C), costa slightly concave, sclerotised; cucullus moderately lobed, membranous, slightly sclerotised, with a central area densely hairy; sacculus internally concave, well sclerotised, distally projected in a finger-like structure, with triangular ventral subdistal process; pulvinus present; vinculum well developed; juxta strongly sclerotised in a rather pentagonal plate; ampulla present; phallus with coecum penis straight, central part strongly curved down, distal part straight, presence of dorsal teeth; vesica with two clusters of non-deciduous cornuti, one proximal oriented ventrally, consisting of aciculate cornuti, basally attached, arranged in a single longitudinal band, another distal consisting of an irregular patch of microspinulate cornuti. Segment 7 in females without modified scaling (corethogyne) but with two lateral, somewhat dorsal pockets on the 7-8 intersegmental membrane. Female genitalia (based on three preparations; Fig. 2F) with sterigma broad; lamella antevaginalis as a simple but evident lobe; ostium in a short funnel like antrum; lamella postvaginalis moderately sclerotised, smooth, broad, with a distinct ventrally prominent distal lobe as a transversal plate; ductus bursae as long as corpus bursae, moderately sclerotised in proximal half, double folded (in Z) when not extended; corpus bursae subspherical, moderately covered internally by acanthae and ctenidia in variable degree of development; no signum or other specially sclerotised area; a long ductus seminalis connected ventrally to cervix, no bulla seminalis; a large globular spermatophore extracted in one of the dissections; anterior apophysis fairly short, projected internally; ventral area of segment 8 behind the sterigma heavily covered by acanthae (spinous field) continuous with distal sclerotised plate of the lamella postvaginalis; a spiny star-shaped ventral subpapillar sclerite on the 8–9 intersegmental membrane at level of ventral lobes of anal papillae (Fig. 4B and D); posterior apophysis simple, approximately same length as papillae; egg pore broad between anal papillae.

#### Biology and distribution.

The early stages are unknown. Adults were collected in January (n = 11) and November (n = 14) at middle elevations (1554-2150 m) in Bolivia, Santa Cruz Department, Florida Province in municipalities of Mairana, El Rasete, and Pampagrande, localities of Agua Clarita, Hueco de la Pascana, and La Hoyada. The collecting sites include transition from dry to cloud forest.

#### Etymology.

The specific epithet refers to the Quechuan word *yun-ka*, which translates as warm valley, a band of forest on the slopes of the Andes Mountains. This zone is of enormous interest from a conservation perspective.

### Brusqeulia araguensis sp. n.

Taxon classificationAnimaliaLepidopteraTortricidae

http://zoobank.org/B86EF700-AFC8-4DF3-9BF2-55DE23D8F8FB

Figures 3 , 4B, D

#### Type material.

***Holotype***: ♂, Venezuela, Aragua State, locality of Rancho Grande, 10°7'N; 67°20.63'W, 10–21 Feb 1969, D. Duckworth and E. Dietz (GS USNM 69274).

***Paratypes***: (4♀). Venezuela, Aragua State, locality of Rancho Grande, 1100 m, 10°7'N; 67°20.63'W, 24-31 Oct 1966 (1♀) (SEM stub JBA202); 22-31 Jul 1967 (3♀), R.W. Poole (GS USNM 85011).

#### Diagnosis.

**Figure 3.**
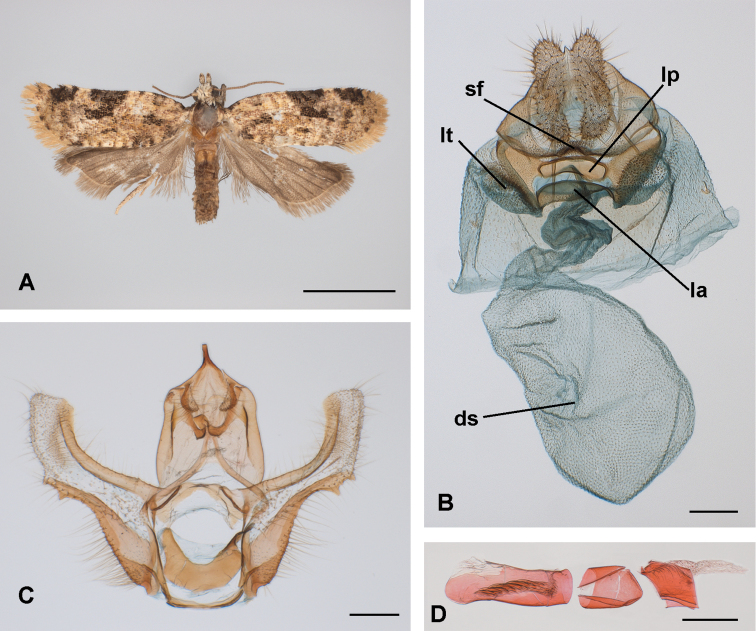
Morphological characters or *Brusqeulia araguensis*. **A** habitus (Paratype, female, Venezuela, Rancho Grande, 22–31 August 1967, USNM) **B** female genitalia (GS USNM85011) **C** male genitalia (GS USNM69274) **D** phallus (fragments photographically assemblage, may not correspond to the real order or orientation) (GS USNM6274). Abbreviations. ds: ductus seminalis connection to the bursa; la, lamella antevaginalis; lp, sclerite on the lamella postvaginalis; lt, lateral pocket; sf, ventral spinous field of segment 8; sp, subpapillar sclerite. Scale bars: 3 mm (**A**); 200 µm (**B, C, D**).

The habitus of *B. araguensis* (Fig. 3A) has more extensive dark brown scaling in the wing pattern compared to *B. yunkensis*, resulting in a more diffuse and ill-defined pattern. A similar pattern is found in *B. teneimorpha* and *B. caracagena*. Species more closely related to *B. yunkensis* (e.g., *B. baeza* and *B. uncicera*) have a more defined, contrasting pattern. The forewing costal crescent-shaped blotch allows clear discrimination between the two species (well defined in *B. yunkensis* and diffuse in *B. araguensis*), but in the context of the genus, these differences could be assumed to represent variation. More diagnostic characters are associated with the male and female genitalia. *Brusqeulia araguensis* can be distinguished by the extremely narrow uncus, the narrowest in the genus, even compare to closely related species such as *B. bonita* and *B. baeza*. The transtilla and gnathos are well developed in *B. araguensis*, similar to most species in *Brusqeulia*, and it is not diagnostic. Teeth or lobes are developed in the distal part of the sacculus coincident with the ventral part of the cucullus in most, if not all, species of the genus. Among congeners, *B. araguensis*, *B. costispina*, and *B. tripuncta* all have several teeth, but their development in *B. araguensis* is moderate compared to the other two species. Finally, the phallus in *B. araguensis* is simpler than in most species of the genus. So far, morphological features of the females of *Brusqeulia* are limited by the paucity of material. The only females available are *B. caracagena*, *B. yunkensis*, and *B. araguensis* (the last two described in this paper). Both share a broad sterigma, but *B. araguensis* and *B. yunkensis* are definitely more closely related to each other than either is to *B. caracagena*, even though differences between them are conspicuous. Both *B. caracagena* and *B. yunkensis* lack the spiny cushion-like asymmetrical areas on the lamella antevaginalis found in *B. araguensis*. *B. caracagena* can be easily distinguished from *B. araguensis* and *B. yunkensis* by the ductus bursae, short in *B. caracagena*, long and convoluted in *B. araguensis* and *B. yunkensis*. The position of the ductus seminalis is clearly different in *B. yunkensis* (from cervix) and *B. araguensis* (from mid-corpus bursae); no information about the ductus seminalis in *B. caracagena* is available. The subpapillar spiny sclerite of the 8–9 intersegmental membrane is pointed in *B. yunkensis* and truncate in *B. araguensis*.

#### Description.

*Head*: Vertex with long whitish scales protruding anteriorly and dorsally, fan-shaped, between antennae. Frons slightly concave covered with a whitish scales. Antenna dark brown, length ca 0.4 as long as forewing costa, dorsally scaled, ventrally ciliated, two rows of scales per flagellomere. Labial palpus porrect, length (all three segments combined) ca. 1.3 times diameter of compound eye, uniformly scaled; first segment short, slightly upcurved with ochreous scales, second segment long, straight with ochreous scales, third segment short, slightly upcurved with a mixed of dominant ochreous scales and a few whitish scales only basally; opening of organ of vom Rath in apical position. Haustellum well developed. Ocelli and chaetosemata well developed.

*Thorax*: Dorsum whitish ochreous with a dorso-apical dark brownish band. Smooth scaled including tegulae, with no tufts. Legs whitish, unmodified, male foreleg hairpencil absent. Forewing length 5.7 mm (n = 1) in males, 5.7–6.2 mm (x̄ = 5.9; n = 4) in females. Forewing pattern (Fig. 3A) not sexually dimorphic. Forewing upperside general background colour whitish with scattered greyish-brown marks; marking ill defined; pairs of strigulae ill defined, concolourous with general background, vaguely detectable, with variable degree of suffusion; basal and subbasal fasciae poorly developed, median fascia as an irregular costal blotch projected tornally, with a small group of dark scales at the level of cubital cell; some coma-like marks on the costa as postmedian and preterminal fasciae; fringe concolourous with general background; forewing underside uniformly brownish ochreous with some pale strigulae on the costa; overlapping area whitish. Hindwing upperside and underside, including fringe, uniformly brownish-ochreous; male costal fold absent; cubital pecten not detected.

**Figure 4.**
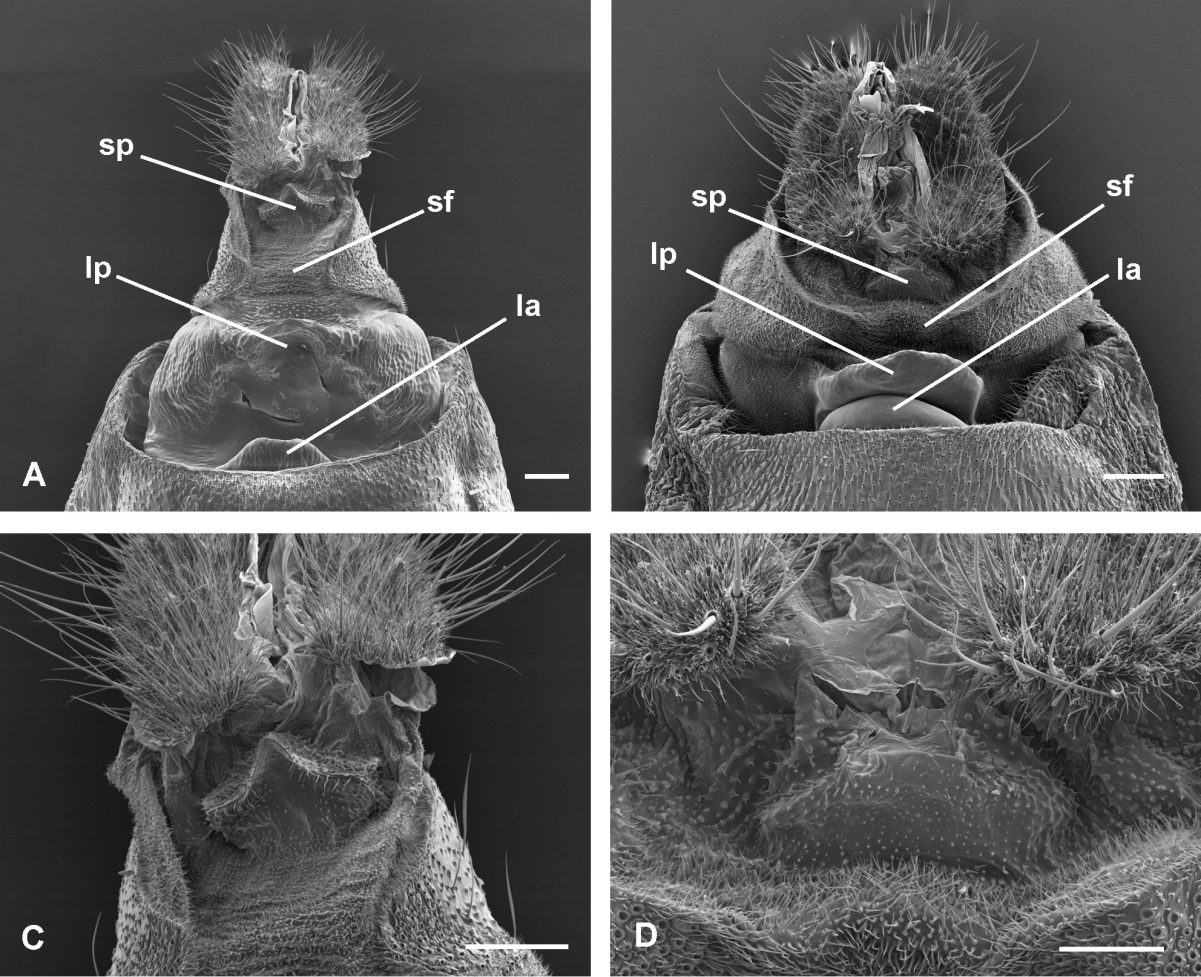
Female terminalia of *Brusqeulia yunkensis* and *B. araguensis*, ventral view, under scanning electron microscopy. **A** *B. yunkensis* **B** *B. araguensis* **C** subpapillar sclerite of *B. yunkensis* **D** same in *B. araguensis*. Abbreviations; la, lamella antevaginalis; lp, lamella postvaginalis; sf, ventral spinous field of segment 8; sp, subpapillar sclerite. Scale bars 100 µm.

*Abdomen*: Dorsally greyish, pale ochreous cephalad. Segment 8 unmodified in males. Male genitalia (based on one preparation; Fig. 3C) with tegumen well developed, laterally straight; uncus slender, straight, basally confluent with top of tegumen to drastically slimmed distally; socii membranous, hairy, obvious, moderately developed; gnathos as two arms distally fused and projected in a short process distally spatulate; transtilla broad, naked; appreciable pulvinus, valva elongate, costa concave, moderately sclerotised, cucullus subrectangular, membranous ventrally, costal area slightly sclerotised, central area densely hairy, sacculus basally convex, distally concave, well sclerotised, transition area of sacculus to cucullus with several tooth like distal process, one of them larger and basal clearly associated to the sacculus, the distal one assignable to the cucullus, a variable number of smaller teeth in between; vinculum broad but rather weakly developed; juxta strongly sclerotised horseshoe shaped; phallus (Fig. 3D) (fragmented in three pieces in the slide) presumably straight with simple caecum, central part broken; no teeth detected on the external surface; vesica simple with two clusters of cornuti, one distal (vesica not evaginated) consisting of non-deciduous (not detected in female corpus bursae) cornuti arranged in a single longitudinal band, another proximal consisting in an irregular patch of microspinulate cornuti. Segment 7 in females without modified scaling (corethogyne) but with two inconspicuous laterodorsal pockets on the 7–8 intersegmental membrane. Female genitalia (based on two preparations; Fig. 3B) with sterigma broad, complex, slightly asymmetrical, ostium simple, slightly on the right; sterigma broad extended laterally in pockets ventrally covered by acanthae continuous laterally with two asymmetrical membranous cushion-shaped areas densely covered by acanthae (Fig. 4A); lamella antevaginalis with a moderately sclerotised convex plate; lamella postvaginalis moderately sclerotised, broad, with a distinct ventrally prominent but smooth dome like plate; ductus bursae rugose, sinuous, posterior half more sclerotised, internally covered by ctenidia continuous with internal vestiture of corpus bursae; corpus bursae subglobular, densely internally covered by ctenidia; no signum or any other sclerotised area detected; ductus seminalis from central area of corpus bursae; no bulla seminalis detected; no spermatophore found; anterior apophysis short projected internally; behind the sterigma the ventral area of segment 8 as a densely spiny lobe; 8–9 intersegmental membrane densely covered by acanthae; densely spiny crescent shape ventral sclerite on the 8–9 intersegmental membrane at the level of the ventral lobes of the anal papillae; posterior apophysis simple, approximately as long as anal papillae; presence of evident broad egg pore between anal papillae.

#### Biology and distribution.

The early stages are unknown. Adults have been collected in February (n = 1), July (n = 2), August (n = 1), and October (n = 1) at middle elevation (1100 m) in Aragua State, Venezuela.

#### Etymology.

The specific epithet refers to the state of Aragua in Venezuela.

## Phylogenetic results

**Figure 5.**
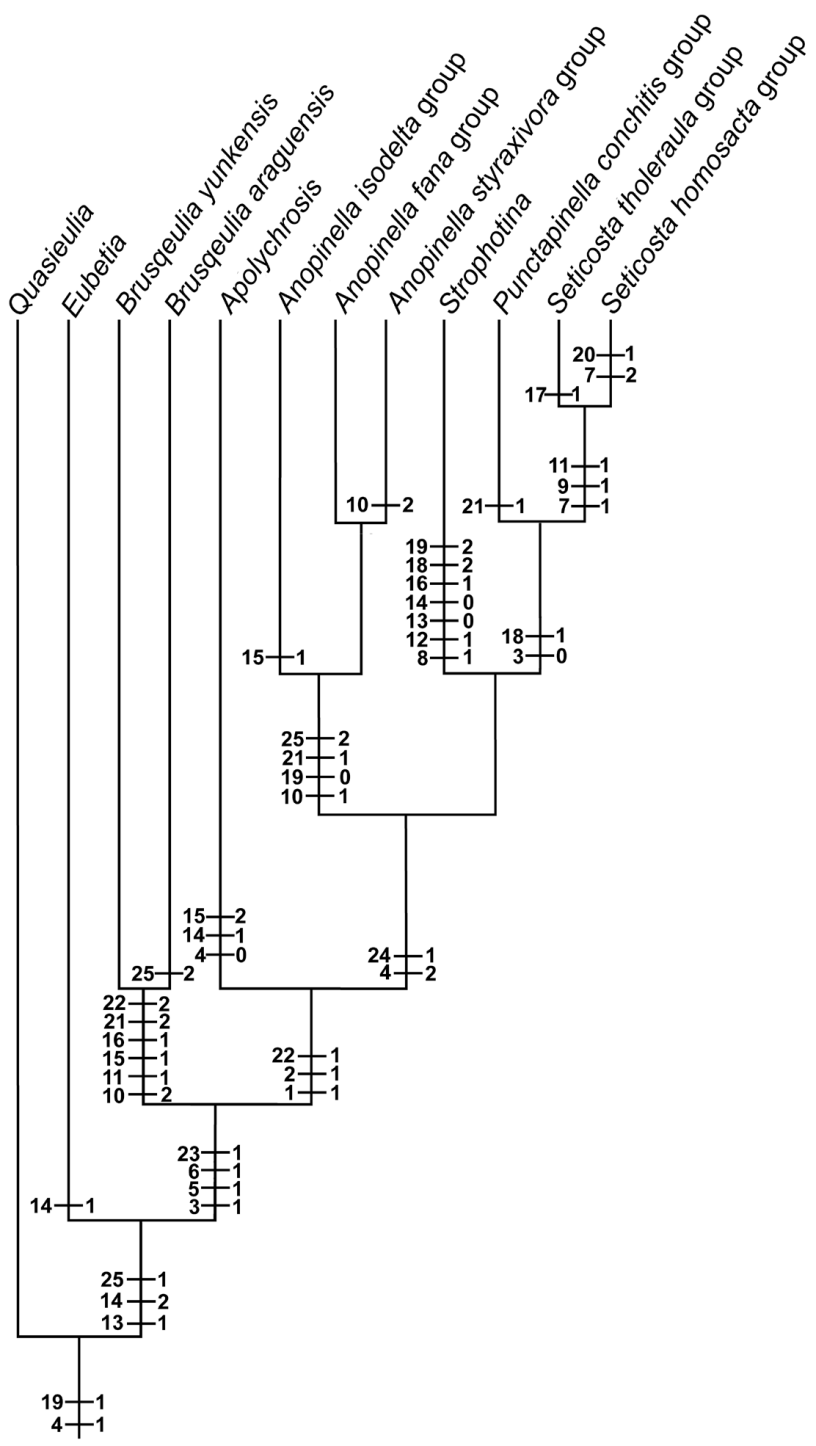
Hypothesis of phylogenetic relationships in the *Apolychrosis* group. *Brusqeulia* is positioned as sister group of the *Apolychrosis* group. The *P. niphastra* group has been excluded from the analysis.

The phylogenetic analysis resulted in five trees of similar topology when using the complete matrix. Small differences are present in the relative position of the *P. niphastra* group with respect to other terminal taxa (the *P. conchitis*, *Seticosta tholeraula*, and *S. homosacta* groups) probably due to the lack of information about the males for the *P. niphastra* group. However, the position of the two new species of *Brusqeulia* remained stable. The consensus trees (including Nelson’s method) rendered a polytomy for the whole *Apolychrosis* group. Nevertheless, removing the *P. niphastra* group from the matrix improved the resolution of the analysis producing a single cladogram (Fig. 5) basically similar to Brown and Adamski (2003). *Brusqeulia* is positioned as sister group of the *Apolychrosis* group in all the analysis.

## Discussion

Based on three species, Razowski and Becker (2000) proposed the following autapomorphies of *Brusqeulia*: a well-developed transtilla, the presence of non-deciduous cornuti, and the configuration of the gnathos. Eleven species were later added (Razowski and Becker 2011) based on males and one more based exclusively on a single female. Although no formal relationships have been proposed, several genera are recognised as having a strong affinity with *Brusqeulia* by Razowski (2016): *Pinhaisania*, *Limeulia*, *Marcelina* Razowski & Becker, 2000, *Saopaulista* Razowski & Becker, 2000, *Crocotaenia* Razowski & Becker, 2003, and *Ibateguara* Razowski & Becker, 2011. All of these share a common appearance and some morphological features of the male genitalia, including the gnathos (with distally joined arms projected into a process) and the valva (with a terminal process in sacculus and a group of slender setae on the cucullus). Among these genera, the relation between *Brusqeulia* and *Pinhaisania* is most conspicuous. Unfortunately, very little information is available about the females of either.

The discovery of two new species including both males and females allows some detailed analysis of characters and rearrangements. The two new species are assigned to *Brusqeulia* on the basis of the characters mentioned above, although *B. yunkensis* could be assigned to *Pinhaisania* based on the conspicuous development of a spinulous transtilla. However, a set of remarkable characters relate *B. yunkensis* to *B. araguensis*, and presumably to other species of *Brusqeulia*, including the absence of CuP in the forewing, two types of non-deciduous cornuti in the vesica of the male genitalia, a characteristic ventral subpapillar sclerotised plate on female segment 9, and the position of the ductus seminalis never associated with the ductus bursae in the female genitalia. Based on this evidence, we propose *Pinhaisania* as a new synonym of *Brusqeulia* and consequently *B. crispula* as a new combination. It would not be surprising if this combination of characters were shared with other genera related to *Brusqeulia* (see above); however, the paucity of material, especially females, does not allow a detailed character analysis.

Among the *Apolychrosis* group of genera (Brown and Adamski 2003), the presence of a characteristic neck in the valva, a subapical process of the sacculus, and the absence of a CuP on the forewing are remarkable defining characters. The two species described in this paper share a robust series of characters consistent with the *Apolychrosis* group of genera. A re-analysis of the character matrix used by Brown and Adamski (2003) including the species here described placed them as a sister group of *Apolychrosis* group.

Molecular evidence already revealed that Cochylina is nested with in Euliina, but the exact point of this connection is still unclear. Our research allows us to suggest a close relationship between Cochylina and the group of genera around *Apolychrosis*. The absence of a CuP on the forewing, the presence of microspinulate areas combined or not with non-deciduous cornuti in the phallus, and a displacement of the ductus seminalis to positions associated to the corpus bursae would be derived characters, all of them putative of Cochylina (Horak and Brown 1991; Horak 1998). A lamella postvaginalis as a central sclerite, and the 8–9 intersegmental membrane covered by acanthae especially remarkable at the sternal level of segment 8 (spinous field by some authors) is also frequent among Cochylina. In *Brusqeulia* there is an additional sclerite just at the ventral base of the anal papillae. A survey of some selected Cochylina did not reveal the presence of this subpapillar sclerite on the female genitalia. However, given the difficult observation of this character and its superficial confluence with the lamella postvaginalis, a more detailed examination of related genera seems necessary to clarify the taxonomic limits of this character.

## Supplementary Material

XML Treatment for Brusqeulia

XML Treatment for Brusqeulia yunkensis

XML Treatment for Brusqeulia araguensis

## Supplementary materials

Supplementary material 1 Material examined Data type: (Data table) Explanation note: Collection data of the voucher material examined. This is an open access article distributed under the terms of the Creative Commons Attribution License (CC BY 4.0), which permits unrestricted use, distribution, and reproduction in any medium, provided the original author and source are credited.Jose V. Pérez Santa-Rita, Joaquin Baixeras

Supplementary material 2 Character matrix Data type: (Phylogenetic matrix) Explanation note: Data matrix for phylogenetic analysis. This is an open access article distributed under the terms of the Creative Commons Attribution License (CC BY 4.0), which permits unrestricted use, distribution, and reproduction in any medium, provided the original author and source are credited.Jose V. Pérez Santa-Rita, Joaquin Baixeras
