# Supplementary material for: Two new species of Brusqeulia Razowski & Becker, 2000 from the Neotropics, with comments on the systematic position of the genus in relation to the Apolychrosis Amsel, 1962 group of genera (Lepidoptera, Tortricidae, Cochylini)
Source: Zookeys. 2018 Jul 3;(770):193–210. doi: 10.3897/zookeys.770.24281 (PMC6041351; doi:10.3897/zookeys.770.24281)
Supplement: Supplementary material 2 — Character matrix [file zookeys-770-193-s002.pdf]

Supplementary file 2: character_matrix

Data matrix for phylogenetic analysis

Apolychrosis	11101	10000	00112	00010	01101
Strophotina	11121	10100	01000	10220	01111
conchitis group	11021	10000	00120	00110	11111
niphastra group	?1?31	1????	?????	?????	?1111
isodelta group	11121	10001	00121	00000	11112
fana group	11121	10001	00120	00000	11112
styraxivora group	11121	10002	00120	00000	11112
*homosact*a group	11021	12010	10120	00111	01111
tholeraula group	11021	11010	10120	01110	01111
Quasieulia	00110	00000	00000	00010	00000
Eubetia	00010	00000	00110	00010	00001
B. yunkensis	00111	10002	10121	10010	22101
B. araguensis	00111	10002	10121	10010	22102
